# Ginsenosides in hematopoietic homeostasis and hematological malignancies: context-dependent pharmacological effects and translational limitations

**DOI:** 10.3389/fphar.2026.1988591

**Published:** 2026-10-16

**Authors:** Jinyu Gai, Bei Zhang, Xinyi Wan, Xiaoling Yu

**Affiliations:** 1 The First Affiliated Hospital of Zhejiang Chinese Medical University (Zhejiang Provincial Hospital of Chinese Medicine), Hangzhou, China; 2 The First Affiliated Hospital of Guangxi University of Chinese Medicine, Nanning, China; 3 The Third Affiliated Hospital of Zhejiang Chinese Medical University, Hangzhou, Zhejiang, China; 4 The Third Clinical Medical College of Zhejiang Chinese Medical University, Hangzhou, Zhejiang, China; 5 Central Laboratory, The First Affiliated Hospital of Zhejiang Chinese Medical University (Zhejiang Provincial Hospital of Chinese Medicine), Hangzhou, China

**Keywords:** applications, bone marrow hematopoiesis, ginsenosides, hematological disorders, leukemia

## Abstract

Ginseng has long been used as a botanical drug in East Asian traditional medical systems, particularly in China and Korea, and as a food ingredient. Ginsenosides are specialized metabolites of *Panax* species with diverse pharmacological activities reported in experimental studies. Evidence regarding their effects in hematological contexts, however, is dispersed across studies using different materials, models, and exposure conditions. This Mini Review synthesizes findings from purified ginsenosides and chemically characterized saponin fractions, focusing on hematopoietic and immune regulation in non-malignant stress models and on malignant hematopoietic cell fate. In non-malignant models, selected metabolites and fractions were associated with reduced cellular injury and changes in peripheral blood, phenotypic, or colony-forming endpoints. In malignant models, effects on proliferation, apoptosis, differentiation, autophagy, and drug transport were reported mainly in cell lines. Clinical evidence remains limited, while gaps in material standardization and pharmacologically relevant exposure constrain translation. By comparing non-malignant and malignant contexts, this review provides an integrated framework for interpreting heterogeneous findings and highlights how metabolite identity, experimental model, cellular context, and exposure may influence the reported effects.

## 1 Introduction

Hematologic disorders constitute a heterogeneous group of diseases affecting the blood, bone marrow, and lymphoid tissues, including leukemias, aplastic anemia, myelodysplastic syndromes, and post-chemoradiotherapy myelosuppression, among others (Kaushansky et al., 2021). Among these, leukemia is a common hematological malignancy in both adults and children, and its treatment continues to face challenges such as relapse, drug resistance, and substantial chemotherapy-related toxicities (Döhner et al., 2015). Meanwhile, chemoradiotherapy-induced myelosuppression is a common dose-limiting toxicity in cancer treatment, potentially compromising treatment delivery and patients’ quality of life (He et al., 2021). Therefore, the development of novel agents that both preserve normal hematopoiesis and inhibit malignant hematologic cells remains a major research priority in the field.

Ginseng refers to the dried roots and rhizomes of *Panax ginseng C. A. Mey.*, a perennial species of the genus *Panax* (family Araliaceae). With a history of medicinal use spanning more than 2,000 years, it has been widely used as a botanical drug in East Asia, particularly in China and South Korea (Park et al., 2012). In traditional Chinese medicine, Dushen Tang, which contains ginseng alone, was used for severe depletion, whereas ginseng-containing formulas such as Renshen Guipi Wan were prescribed for patterns described as qi and blood deficiency (Yang et al., 2019; Chinese Pharmacopoeia Commission, 2025). *Panax notoginseng* (Burkill) F.H.Chen has traditionally been used for bleeding-related conditions (Wang et al., 2016). Steamed P. notoginseng has also been investigated in a cyclophosphamide/acetylphenylhydrazine-induced “blood deficiency” model, while comparative processing studies indicate that steaming and frying alter its saponin profile and reported hematological endpoints (Zhang et al., 2019; Xu et al., 2024). The traditional construct of xue xu should not be equated directly with anemia, aplastic anemia, or drug-induced myelosuppression. These practices provide a rationale for investigating Panax-derived materials in hematopoietic and immune contexts.

Ginsenosides are major specialized metabolites of *Panax* species and are classified primarily into dammarane-type saponins, including protopanaxadiol (PPD), protopanaxatriol (PPT), and ocotillol types, and oleanane-type saponins (Ru et al., 2015; Piao et al., 2020; Shi et al., 2019). Representative PPD-type metabolites include Rb1, Rb2, Rc, Rd, and Rg3, whereas PPT-type metabolites include Rg1, Rg2, and Rh1. Pseudoginsenoside F11 and ginsenoside Ro represent ocotillol- and oleanane-type metabolites, respectively (Shi et al., 2019). Ginsenoside profiles vary according to *Panax* species, plant part, geographical origin, and processing conditions (Li et al., 2025; Shi et al., 2022; Lee et al., 2018; Zeng et al., 2026; Yun et al., 2025). In particular, steaming can convert relatively polar parent ginsenosides into less-polar metabolites, including Rg3, Rg5, Rk1, Rk3, Rh1, and Rh4, thereby altering the chemical profile of the preparation (Lee et al., 2018; Zeng et al., 2026; Heinrich et al., 2022).

Traditional decoctions and processed botanical drugs are complex mixtures and are not pharmacologically interchangeable with purified ginsenosides or chemically defined saponin fractions (Heinrich et al., 2022). The latter materials permit more reproducible dosing and more specific attribution of observed effects. Accordingly, this review focuses on purified ginsenosides and chemically characterized saponin fractions. Material identity, source, purity, batch information, botanical authentication, and chromatographic characterization are considered when evaluating the reliability of the included evidence.

Ginsenosides have been associated with immunomodulatory, antitumor, antioxidant, and anti-inflammatory activities in experimental studies (Tong et al., 2022). Previous reviews have summarized the antileukemic findings reported for selected ginsenosides, including Rg3, Rh2, and Rg1 (Dana et al., 2024), or their effects in hematopoietic injury and myelosuppression models (He et al., 2021). However, these reviews have provided limited integration of evidence across non-malignant and malignant hematopoietic contexts. This Mini Review therefore evaluates ginsenosides as potential context-dependent pharmacological modulators. It compares evidence from non-malignant hematopoietic stress and malignant hematopoietic models and examines how metabolite identity, exposure, cellular state, and disease context may contribute to the heterogeneous findings.

Unlike previous reviews centered on individual ginsenosides or specific hematological conditions, this Mini Review compares evidence across non-malignant hematopoietic stress and malignant hematopoietic models within a common analytical framework. Each finding is evaluated according to material identity and characterization, experimental context, dose or exposure, evidence tier, mechanistic validation, and principal limitation. This approach distinguishes observed endpoints from causal inference and examines whether the reported protection of non-malignant hematopoiesis and suppression of malignant cells constitute a defensible context-dependent pattern.

PubMed, Web of Science, Scopus, and the Cochrane Library were searched from database inception to September 2026. Google Scholar and reference lists were used for supplementary citation tracking. Search terms combined ginsenoside- or *Panax*-related terms with hematopoiesis, bone marrow failure, myelosuppression, anemia, leukemia, pharmacokinetics, and safety. Cellular, animal, and clinical studies of purified ginsenoside metabolites, sapogenins, and chemically characterized saponin preparations were considered. Crude whole-plant extracts and multi-herbal formulations were excluded from the core evidence synthesis. Studies prioritized for the main evidence synthesis are summarized in Table 1 according to material identity, hematopoietic context, experimental model, exposure, evidence tier, mechanistic validation, and principal limitation. Metabolite/material and characterization Biological context, Model, Exposure, Pharmacological outcome/mechanistic support, Evidence tier, Key exposure or design limitation, Study (current reference).

**TABLE 1:** Summarize the metabolite or preparation, biological context, model, exposure, evidence tier, and principal limitation of the studies prioritized in the main text.

Context-dependent pharmacological effects of ginsenosides in non-malignant and malignant hematopoietic models							
Metabolite/material and characterization	Biological context	Model	Exposure	Pharmacological outcome/mechanistic support	Evidence tier	Key exposure or design limitation	Study (current reference)
Rg1 (purified monomer; 96–>98% purity)	Normal/protective: immune-mediated AA and stress-associated HSPC aging	Irradiation plus DBA/2 lymphocyte-transfer AA mice and LSK cells; t-BHP-treated murine Sca-1+ cells with non-competitive serial transplantation	AA: 20–80 mg/kg/day *in vivo*; 12.5–50 μM *ex vivo*. Aging/transplantation: 10 μM *ex vivo*; 20 mg/kg/day *in vivo*	Improved blood/marrow or colony-forming endpoints and reduced mitochondrial apoptosis; serial transplantation supported persistence of selected aging-related endpoints	Tier 2 + Tier 3	LSK or Sca-1 alone does not define long-term HSCs; transplantation was non-competitive and did not quantify durable multilineage donor chimerism; pathway evidence was largely associative	Cao et al. (2021); Zhou et al. (2015)
Rg1 (purified monomer; ≥95% purity)	Normal/protective: radiation- or CTX-associated hematopoietic stress	6.5-Gy irradiated mice and Sca-1+ HSC/HPCs; CTX-treated mice with splenectomy or sham surgery	20 mg/kg/day for 7 days before irradiation; 15 mg/kg/day intraperitoneally for 7 days after CTX	Reduced senescence/oxidative-stress phenotypes; splenectomy abolished Rg1-associated increases in circulating c-Kit+CD45^+^ and marrow LSK cells, implicating splenic extramedullary hematopoiesis	Tier 3 (organ-level perturbation in one study)	Predominantly preventive or acute injury models; no cell tracing, competitive transplantation, long-term self-renewal assessment, PK, or marrow exposure measurement	Tang et al., (2020); Liu et al. (2015)
PDS and PTS (defined saponin fractions; PDS major saponins 91.64%, PTS 75.46%; chromatographically analysed)	Normal/protective: chemotherapy-induced myelosuppression	CTX-treated mice	0.10 g/kg orally for 28 days; CTX 50 mg/kg on days 25–28	Improved selected peripheral counts, marrow cell-cycle/apoptosis indices, and immune readouts; mechanistic support was based on associated protein and cytokine changes	Tier 3	Preventive design, multicomponent fractions, no causal rescue, long-term HSC transplantation, PK, or marrow exposure data	Zhang et al. (2022)
LPNS (defined P. notoginseng leaf-saponin fraction; 79.22% total saponins; fingerprint/MS characterization)	Normal/protective: immune-mediated marrow failure	Irradiation plus lymphocyte-transfer murine AA model	50, 100, or 200 mg/kg	Improved hematological and progenitor-cell endpoints and shifted Th1/Th17 versus Th2/Treg proportions; findings support immunomodulation rather than long-term HSC restoration	Tier 3	Multicomponent fraction; short-term animal model; randomization/blinding, causal immune perturbation, transplantation, PK, and marrow exposure were not established	Zhao et al. (2018)
Panaxadiol saponins (PND/PDS fraction; PND20220912: >88% total purity, Rb1 30%–35%, Rb2 20%–25%, Rc 15%–20%; HPLC–MS)	Normal/protective: immune-mediated AA, inflammatory microenvironment, and iron-overload/ferroptosis context	Immune-mediated AA mice, primary murine BMNCs/colony assays, LPS injury; iron-overload AA mice and Meg-01 cells	Generally 20–80 mg/kg/day in mice; 20–80 μg/mL or up to 160 mg/L *ex vivo*; Meg-01, 20 mg/L	Improved blood/marrow and immune endpoints and reduced NLRP3- or ferroptosis-associated signals; MCC950 and CETSA provided limited target/pathway support	Tier 1 + Tier 2 + Tier 3	The 2023 preparation cannot be assumed identical to the later standardized batch; microbiota and target claims remain incompletely causal; high exposures, no human PK/marrow exposure, and no long-term HSC testing	Liu et al. (2023); Mi et al. (2027)
Re and Rk3 (purified monomers; ≥98%, HPLC confirmed)	Normal/protective: CTX-induced myelosuppression	CTX-treated BALB/c mice and *ex vivo* marrow colony assays	5 or 10 mg/kg intraperitoneally for 7 days after CTX	Partly corrected blood/marrow endpoints and increased CFU-E, BFU-E, CFU-GM, and CFU-Meg; several outcomes favoured Rk3 over Re	Tier 3	Acute drug-induced myelosuppression, not acquired AA; no phenotypic/functional HSC transplantation, causal pathway intervention, PK, or marrow exposure; spontaneous recovery complicates interpretation	Han et al. (2019)
Total ginsenosides (defined mixture: Rg1, Re, Rf, Rg2, Rb1, Rc, Rb2, and Rd quantified)	Context comparison: erythroid differentiation in malignant and normal progenitors	K562 cells, normal human CD34^+^ erythroid progenitors, and Townes β-thalassemia mice	25–400 μg/mL *in vitro*; 50–800 mg/kg/day in mice	Increased γ-globin/HbF-related endpoints across models, providing a normal-versus-malignant cellular comparison rather than evidence of general hematopoietic restoration	Tier 1 + Tier 2 + Tier 3	Multicomponent preparation, heterogeneous models, high animal doses, uncertain active metabolite(s), no PK/exposure bridge, and no clinical evidence	Cai et al. (2025)
Pai-Neng-Da capsule (PNDC; panaxadiol-saponin preparation; batch 20150401; reported HPLC purity 92.44%)	Clinical: chronic aplastic anemia	Multicentre randomized comparative study; 79 randomized and 78 evaluable patients	PNDC plus cyclosporine and reduced-dose testosterone undecanoate for 24 weeks versus cyclosporine plus full-dose testosterone undecanoate	Protocol-defined response was 88.1% versus 77.8% (P > 0.05), with comparable endpoint blood counts; female masculinization scores were lower in the PNDC/reduced-androgen group	Tier 4	Small sample; blinding/allocation concealment and prospective registration incompletely reported; androgen dose differed concurrently; individual saponin composition was not fully reported, preventing attribution of independent benefit to PNDC.	Jiang et al. (2022)
Compound K (CK; purified metabolite)	Malignant/cytotoxic: AML	U937, THP-1, MV4-11, C1498, MOLM-13, and NB4 cells; C1498/FLT3 murine leukemia model	10–40 μM *in vitro*; 1 or 5 mg/kg *in vivo*	Induced G1 arrest/apoptosis and reduced leukemia burden; transcript/protein changes included lower BCL2, KIT, DNMT3A, MYC, and CSF1 and higher TET2	Tier 1 + Tier 3	No normal CD34^+^ HSPC, stromal-cell, or primary-patient comparator; no causal rescue for most targets; clinically attainable exposure and marrow concentrations are unknown	Hou et al. (2022)
20(S)-PPD (purified metabolite; stereochemistry specified; complete batch/QC reporting inconsistent)	Malignant/cytotoxic: AML and venetoclax sensitization	Multiple AML cell lines and an animal model; separate study included healthy-donor PBMCs	Approximately 20–60 μM *in vitro*; animal exposure reported in the *in vivo* study	Suppressed PI3K/AKT/mTOR signaling and activated sustained PERK/ATF4/CHOP-associated stress; Bax/Bak knockdown and MCL-1/Bcl-XL overexpression partly rescued apoptosis in a separate study	Tier 1 + Tier 2 + Tier 3	Active concentrations were high; PBMCs do not establish safety in CD34^+^ HSPCs or marrow stroma; limited primary AML data and no human PK or marrow-exposure bridge	Zhang et al. (2025); Xu et al. (2025)
24-OH-PD (purified metabolite; reported HPLC–ELSD purity 99.87%)	Malignant/cytotoxic: T-ALL	CCRF-CEM cells and an animal model	60–80 μM *in vitro*; 1 or 10 mg/kg *in vivo*	Induced ROS-associated mitochondrial apoptosis and reduced T-ALL growth in the experimental models	Tier 1 + Tier 3	Single principal cell-line context; no normal HSPC, marrow-stromal, or primary-patient comparator; high *in vitro* concentrations and clinical attainability are unresolved	Li et al. (2023)
20(S)-Rh2 (purified metabolite; 95%, CAS 78214-33-2)	Malignant/context-dependent: ATRA-resistant APL	NB4-R1/NB4-R2 cells and subcutaneous xenograft models	Exposure requires final full-text verification	Reduced lactylation/HDAC-associated signaling and modulated METTL3, with increased apoptosis and restored ATRA responsiveness in resistant models	Tier 1 + Tier 3	No normal HSPC or primary-patient comparator; subcutaneous models do not reproduce marrow disease; interaction with ATRA, systemic exposure, and clinical attainability remain uncertain	Cheng et al. (2024)
Rk3 (purified monomer; >98%, batch 140606; structural identification reported)	Malignant/microenvironmental: monocytic AML and extramedullary infiltration	SHI-1 cells and patient-derived cells	60–180 μmol/L	Reduced proliferation, migration, and invasion; overexpression and reporter experiments supported involvement of the miR-3677-5p/CXCL12/CXCR4 axis	Tier 1 + Tier 2	High concentrations; limited patient-cell details; no normal HSPC/stromal comparator, orthotopic *in vivo* validation, PK, or marrow/extramedullary tissue exposure	Ma et al. (2022)
20(R)-/20(S)-Rg3, 20(S)-PPD, and 20(S)-PPT (stereoisomers/metabolites distinguished; source, purity, batch, and chromatography incompletely reported)	Malignant/resistance: P-gp-associated multidrug-resistant leukemia	CCRF-CEM parental and CEM/VLB1000 resistant cells; single-cell biochip daunorubicin-uptake assay	Concentrations require final full-text verification	Increased intracellular daunorubicin in resistant cells, consistent with modulation of drug efflux; comparative activity varied among structurally related metabolites	Tier 1	Single *in vitro* resistance model; transporter binding/inhibition and downstream survival were not fully established; increased drug uptake may also increase normal-tissue toxicity; no PK or clinical validation	Chen et al. (2026)
Rd (purified monomer; >98%, CAS 52705-93-8)	Malignant/differentiation: myeloid leukemia	KG-1, K562, HEL, and NB4 cells; subcutaneous K562 xenografts	25–200 μg/mL *in vitro*; 200 mg/kg/day for 4 weeks *in vivo*	Reduced viability/colony formation and altered differentiation markers; ERK inhibition partly reversed selected POX and GATA-1/PU.1 changes	Tier 1 + Tier 3	High exposures; K562 is not AML; subcutaneous xenografts do not reproduce marrow leukemia; no normal HSPC or primary-patient comparator, PK, marrow exposure, or direct GSK-3β causality	Jiang et al. (2024)
20(S)-Rh2 (purified metabolite; 98%)	Malignant/context-dependent: AML apoptosis and differentiation	HL-60 and Kasumi-1 cells; disseminated HL-60/NOD-SCID model	5–100 μM *in vitro*; 20 mg/kg/day orally for 3 weeks	Induced apoptosis and differentiation-associated phenotypes; Nur77 knockdown attenuated apoptosis, death-receptor expression, and differentiation readouts, supporting a causal role for Nur77	Tier 1 + Tier 3	No normal HSPC, stroma, or primary AML comparator; small animal groups and no quantitative leukemia burden/survival endpoint; active concentrations, PK, and marrow exposure remain unresolved	Wang et al. (2017)
KBB-N1/compound K (purified metabolite; >95%)	Malignant/cytotoxic with normal-progenitor comparison: multiple myeloma	ARH77, U266, and RPMI8226 cells; healthy-donor progenitor colonies; NSG xenografts; rat PK	20–70 μM *in vitro*; 60 μM progenitor assay; 20 mg/kg/day orally or 7 mg/kg IV in mice; 10 mg/kg oral/IV rat PK	Induced apoptosis/sub-G1 accumulation and prolonged xenograft survival; healthy-donor colonies remained detectable at 60 μM	Tier 1 + Tier 2 + Tier 3	Selectivity was not quantified; p53 involvement lacked causal validation; oral rat Cmax (∼0.61 μM) was below the 20–70 μM active range; no marrow exposure or primary myeloma cells	Lee et al. (2024)
20(S)-Rh2 (purified metabolite; 99.48%)	Malignant/context-dependent: ALL apoptosis versus cytoprotective autophagy	Reh and Jurkat cells; six patient marrow samples; healthy-donor B/T cells	20–60 μM for 24 h; mainly 40 μM for mechanistic experiments	Induced mitochondrial apoptosis and autophagy; pharmacological inhibition and ATG5 knockdown enhanced apoptosis, supporting cytoprotective autophagy; healthy-donor B/T cell viability was not significantly reduced under tested conditions	Tier 1 + Tier 2	Small patient/control samples; no CD34^+^ HSPC or marrow-stromal comparator; no animal/PK evidence; no p62 turnover or tandem-fluorescent flux reporter; therapeutic window cannot be defined	Xia et al. (2016)
Rg3 preparation (mainly Shenyi capsule) plus chemotherapy	Clinical/indirect: chemotherapy-induced myelosuppression in patients with solid tumours	Systematic review/meta-analysis of 18 RCTs (2,222 patients)	Usually 20 mg twice daily; 3–4-week cycles, generally two or four cycles	Associated with lower risks of leukocyte, hemoglobin, platelet, and neutrophil decline versus chemotherapy alone; authors judged the underlying trials methodologically weak	Tier 5	Mostly Chinese single-centre trials; allocation concealment/blinding often unclear; heterogeneous chemotherapy regimens and incomplete product standardization; indirect for primary hematological disorders and not proof of ginsenoside-specific clinical benefit	Pan et al. (2020)
Red/fermented red-ginseng preparations (human PK) and BST204 (defined Rh2/Rg3-rich fraction; rat stereoselective PK)	Translational determinant: gut-microbiota variability and stereoisomer-specific exposure	34 healthy volunteers with oral red-ginseng preparations; rats given BST204 or purified 20(S)-Rh2/20(S)-Rg3	Human oral doses as reported for each preparation; BST204 400–2,000 mg/kg orally; purified 20(S)-Rh2 31.5 mg/kg and 20(S)-Rg3 68 mg/kg	Fecal CK-forming activity correlated with serum CK AUC (r = 0.566); in rats, S-epimers predominated in plasma/tissues, with distribution mainly to liver and gastrointestinal tract	Tier 4 (human PK) + Tier 3 (animal PK)	Human association does not prove microbial causality; preparation-specific findings cannot be generalized to purified metabolites; animal doses were high; neither study established bone-marrow exposure or attainment of leukemia-cell active concentrations	Kim et al. (2020); Bae et al. (2014)
Interpretation notes							
Evidence hierarchy			Tier 1, established cell lines; Tier 2, primary human or animal cells/patient-derived samples; Tier 3, animal models (specific perturbation/rescue is stated where present); Tier 4, clinical or human PK evidence; Tier 5, systematic review/meta-analysis				

Material characteristics of the included interventions are summarized in Supplementary Table S3.

## 2 Ginsenosides as regulators of hematopoietic and immune homeostasis

Hematopoietic homeostasis depends on the coordinated regulation of HSPC self-renewal, survival, lineage differentiation, and signals from the bone marrow microenvironment (Dzierzak and Bigas, 2018; Fleming et al., 2008). In experimental stress models, selected ginsenosides were associated with changes in phenotypically defined HSPC populations, colony-forming activity, apoptosis-related endpoints, and peripheral blood counts (Wang et al., 2022; Cao et al., 2021; Wang et al., 2024; Han et al., 2019; Zhou et al., 2015).

### 2.1 Regulation of hematopoietic stem/progenitor cells

#### 2.1.1 Effects on HSPC-associated recovery and progenitor output

HSCs sustain lifelong hematopoiesis through self-renewal and multilineage differentiation, whereas more committed progenitors provide short-term blood-cell production (Dzierzak and Bigas, 2018; Fleming et al., 2008). In an immune-mediated murine aplastic-anemia model, purified Rg1 increased LSK-cell abundance and erythroid colony formation and reduced ROS accumulation, mitochondrial Bax translocation, and apoptosis (Cao et al., 2021). Rg1 also reduced senescence-associated endpoints and increased colony formation in oxidatively stressed murine Sca-1^+^ marrow cells; noncompetitive serial transplantation supported persistence of selected protective effects, but durable multilineage donor reconstitution was not demonstrated (Zhou et al., 2015).

Myelosuppression is a common dose-limiting toxicity of chemotherapy and radiotherapy and may compromise treatment delivery and quality of life (Brigle et al., 2017; Zhang et al., 2024). More than one-third of patients undergoing chemotherapy develop cytopenia and reduced bone marrow cellularity as adverse effects. In CTX-induced murine myelosuppression, purified Re and Rk3 partially improved peripheral-blood and bone-marrow-cell indices and increased *ex vivo* progenitor colony formation, with several endpoints favoring Rk3 (Han et al., 2019). In the same CTX-induced myelosuppression context, a UPLC/IMS-QTOF-profiled total ginsenoside fraction was associated with increased expression of Notch1, Notch2, Jag1, Wnt7b, Wnt10b, and Fzd6 and decreased Hes1 expression in bone-marrow HSC-enriched cells (Gao et al., 2025). Because neither pathway perturbation nor transplantation was performed, these findings indicate changes in Notch- and Wnt-related markers rather than pathway-dependent restoration of long-term HSC function. Following 6.5-Gy total-body irradiation, Rg1 pretreatment increased SOD2 expression and improved senescence and colony-forming endpoints in Sca-1^+^ HSPC-enriched cells (Tang et al., 2020). However, SOD2 activity, competitive transplantation, and long-term multilineage reconstitution were not assessed.

At the lineage-specific level, an HPLC-characterized total ginsenoside preparation increased γ-globin expression and HbF-related endpoints in K562 cells, normal cord-blood-derived CD34^+^ erythroid progenitors, and human-globin transgenic mice (Cai et al., 2025). This cross-model consistency supports an effect on erythroid differentiation, but the high exposures and multi-metabolite preparation preclude attribution to an individual metabolite, and clinical benefit in β-thalassemia has not been established.

Collectively, these studies support effects on short-term progenitor activity, oxidative-stress responses, and hematopoietic recovery in experimental injury models. However, most pathway evidence is based on associated changes in gene or protein expression. None of the studies performed competitive transplantation, and the available noncompetitive serial-transplantation data did not establish durable multilineage reconstitution. The evidence therefore does not demonstrate restoration of long-term HSC self-renewal.Additional *ex vivo* and short-term animal studies are summarized in Supplementary Table S1.

#### 2.1.2 Modulation of hematopoietic microenvironments

After birth, the bone marrow is the principal site of hematopoiesis, whereas hematopoietic stress may induce extramedullary hematopoiesis. Overlapping endosteal and perivascular signals regulate HSC quiescence, survival, and lineage output (Kwon et al., 2024; Nakamura et al., 2010). In D-galactose-induced senescence models, purified Rg1 (>99.63%, 40 mg/kg/day in mice and 50 μM *in vitro*) reduced inflammatory signaling in mesenchymal stromal cells and increased the colony-forming activity of cocultured murine bone marrow cells. Attenuation of these effects by a TLR1/2 agonist supported the involvement of TLR2/NF-κB signaling, although direct target engagement and long-term HSC function were not established (Wei et al., 2025). In CTX-treated mice, Rg1 (≥95%, 15 mg/kg/day, intraperitoneally) attenuated splenic extramedullary hematopoiesis and increased circulating c-Kit^+^CD45^+^ cells and bone marrow LSK cells. These increases were absent after splenectomy, indicating dependence on the spleen; however, cell migration, niche-cell interactions, and long-term repopulating activity were not directly examined (Liu et al., 2015). Thus, current evidence supports modulation of stromal inflammatory responses and spleen-associated hematopoietic recovery, rather than structural rejuvenation of the HSC niche.

### 2.2 Modulation of inflammatory and immune microenvironments

While exerting cytotoxic effects against tumor cells, chemotherapy agents (e.g., cyclophosphamide) also inflict damage on normal hematopoietic tissue and the immune system. Furthermore, chemotherapy-induced gut microbiota dysbiosis exacerbates systemic inflammation and myelosuppression. Pro-inflammatory cytokines induced during radiation therapy and vascular endothelial damage can further impair hematopoietic homeostasis and compromise immune function (Shao et al., 2014). In CTX-treated mice, chemically characterized PDS and PTS fractions partially normalized selected serum cytokines, bone-marrow cell-cycle distribution, and apoptosis-related endpoints (Zhang et al., 2022). However, cytokines were measured in serum and cell-cycle and apoptosis endpoints in bulk bone-marrow cells; Th1/Th2 populations and pathway dependence were not directly examined. In a dexamethasone-induced immunosuppression model, an HPLC-characterized black-ginseng total-saponin fraction increased immune-organ indices, immunoglobulins, and several cytokines and was accompanied by increased hepatic TLR4/MyD88/NF-κB protein expression (Wang et al., 2026). These findings do not establish pathway causality or identify the contributions of individual metabolites, and the concurrent increases in IL-1β and TNF-α complicate interpretation as uniformly beneficial immune enhancement. In CTX-immunosuppressed mice, oral PPD increased phenotypically defined bone-marrow HSPCs and selected immune indices, whereas these changes were largely absent after gut-microbiota depletion (Cao et al., 2020). This intervention supports a contribution from the microbiota, although the responsible taxa, microbial metabolites, and gut-to-marrow mechanisms remain undefined.

Acquired aplastic anemia (AA) is predominantly an immune-mediated bone-marrow-failure disorder (Cree, 2022). In an irradiation-assisted lymph-node-cell-transfer model of AA, a chemically characterized Panax notoginseng leaf-saponin fraction improved peripheral-blood and colony-forming endpoints and shifted splenic T-cell profiles from Th1/Th17- toward Th2/Treg-associated responses (Zhao et al., 2018). In another immune-mediated AA model, standardized PND (>88% total purity; enriched in Rb1, Rb2, and Rc) improved selected blood-count and CD4^+^/CD8^+^ endpoints and reduced NLRP3-associated signaling. MCC950 experiments in primary bone-marrow nucleated cells provided pharmacological support for NLRP3 involvement, although microbiota-mediated causality was not established (Mi et al., 2027). An earlier panaxadiol-saponin preparation also altered peripheral T-cell subsets and reduced serum TNF-α, IL-6, and IFN-γ in an iron-overload AA model (Liu et al., 2023). However, its ferroptosis-related findings were partly obtained in malignant Meg-01 cells and cannot be extrapolated to normal megakaryocytes. Overall, these animal studies support short-term immunological and progenitor-associated effects, but they do not establish long-term HSC recovery or clinical benefit.

### 2.3 Modulation of malignant hematopoietic cell fate

Hematological malignancies are characterized by dysregulated proliferation, impaired differentiation, and resistance to cell death (Egan and Schimmer, 2023). Experimental studies suggest that several ginsenosides affect these processes, but the evidence is predominantly derived from cell lines and animal models. Normal CD34^+^ HSPCs, bone-marrow stromal cells, and primary patient samples were rarely included, limiting assessment of malignant-cell selectivity.

#### 2.3.1 Apoptosis, differentiation, and autophagy in malignant cells

In AML cell lines, compound K (20–40 μM) reduced viability and colony formation and induced apoptosis and G1-phase accumulation, accompanied by decreased *BCL2* mRNA expression (Hou et al., 2022). However, primary blasts and normal hematopoietic comparators were not examined. In AML cell lines and primary AML samples, 20(S)-PPD reduced viability and induced apoptosis, accompanied by decreased AKT/S6 signaling and activation of the eIF2α/ATF4 stress response. Partial attenuation by ISRIB supported involvement of the integrated stress response (Zhang et al., 2025). A separate study demonstrated Bax/Bak-dependent apoptosis associated with MCL-1 and Bcl-XL downregulation and enhanced venetoclax activity *in vitro* (Xu et al., 2025). Healthy-donor PBMCs were less affected under the tested conditions, but these findings do not establish selectivity relative to normal CD34^+^ HSPCs.

Differentiation-associated effects have also been reported. Purified Rd induced morphological and immunophenotypic differentiation in myeloid leukemia cell lines, and ERK inhibition partially reversed selected differentiation-associated changes (Jiang et al., 2024). In HL-60 and Kasumi-1 AML cells, 20(S)-Rh2 induced apoptosis and differentiation-associated phenotypes that were attenuated by Nur77 knockdown. Oral treatment also reduced disease-associated endpoints in an HL-60/NOD-SCID model (Wang et al., 2017). Rh2-induced myeloid differentiation in HL-60 cells was attenuated by TGF-β1 neutralization (Chung et al., 2013). Compound K increased megakaryocytic markers in K562 and Meg-01 cells (Hwang et al., 2024). However, differentiation of malignant cell lines does not establish increased thrombopoiesis or preserved platelet function in normal hematopoiesis.

Autophagy may attenuate the cytotoxic response to 20(S)-Rh2. In U937 and K562 cells, pharmacological autophagy inhibition enhanced Rh2-induced apoptosis, although autophagic flux was not directly measured (Zhuang et al., 2018). More direct evidence was obtained in ALL cell lines and primary ALL cells, in which 20(S)-Rh2 induced autophagic flux and mitochondrial apoptosis. In the same study, ATG5 knockdown and pharmacological autophagy inhibition further increased apoptosis, supporting a cytoprotective role for autophagy (Xia et al., 2016). Healthy-donor B- and T cell viability was less affected, but normal CD34^+^ HSPCs were not evaluated, and whether the tested exposures are clinically attainable remains uncertain.

In T-ALL models, chemically characterized 24-OH-PD induced ROS-associated mitochondrial apoptosis in CCRF-CEM cells and prolonged survival in a corresponding xenograft model (Li et al., 2023). In ATRA-resistant APL models, 20(S)-Rh2 reduced lactylation- and METTL3-associated signaling and enhanced ATRA-induced differentiation *in vitro* and in xenografts (Cheng et al., 2024). However, primary APL cells and normal hematopoietic comparators were not included, and molecular docking alone does not establish direct Rh2–METTL3 binding. In Jurkat T-ALL and Reh B-ALL cells, purified 20(S)-Rh2 (99.48% purity, 35 μM) reduced viability and induced cell-cycle arrest and apoptosis, accompanied by decreased PI3K/AKT/mTOR phosphorylation. Treatment at 40 mg/kg also reduced leukemic burden in a CTX-conditioned Jurkat xenograft model (Xia et al., 2020a). However, pathway dependence, selectivity relative to normal CD34^+^ HSPCs, and the clinical attainability of the tested exposures were not established.

KBB-N1 (compound K, purity >95%) induced apoptosis in myeloma cells and prolonged survival in an NSG xenograft model. Healthy-donor progenitor colonies remained detectable at 60 μM, but quantitative selectivity was not established, and reported oral systemic exposure was below the *in vitro* active range (Lee et al., 2024).

Overall, these findings support context-dependent cytotoxic and differentiation-associated effects, but they do not define a therapeutic window because normal HSPC testing and pharmacologically relevant exposure data remain insufficient.

#### 2.3.2 Stem-like leukemia-cell phenotypes, host context, and drug resistance

The tumor microenvironment, composed of stromal cells, endothelial cells, immune cells, and soluble factors, forms a dynamic ecosystem that is critical for the survival, progression, and immune evasion of leukemic cells in AML (Hanahan and Coussens, 2012). During leukemogenesis, LSCs can remodel the tumor microenvironment, thereby promoting their own proliferation and survival (Sendker et al., 2021). However, direct evidence that ginsenosides disrupt these interactions remains limited. KG1α is an AML-derived cell line with a CD34^+^CD38^−^ phenotype commonly used for LSC enrichment, but this phenotype alone does not establish functional stem-cell activity (Chan and Huntly, 2008). In CD34^+^CD38^−^ KG1α cells, Rg1 increased senescence-associated changes, accompanied by increased p16^INK4a^ and decreased hTERT expression (Tang et al., 2021). However, the use of an LSC-enriched cell line without primary AML cells or functional transplantation assays precludes conclusions regarding selective targeting of LSCs.

Host-associated effects have also been reported. In CTX-conditioned NOD/SCID mice bearing Jurkat T-ALL xenografts, oral 20(S)-Rh2 reduced leukemic burden and was associated with lower PI3K/AKT/mTOR phosphorylation, altered splenic immune indices, improved intestinal-barrier endpoints, and changes in gut-microbiota composition (Xia et al., 2020b). However, CTX conditioning may itself affect immune and intestinal endpoints, and the contribution of the microbiota or barrier changes to the antileukemic response was not tested by depletion, transfer, or rescue experiments.

In SHI-1 AML cells, purified Rk3 (>98%; 60–180 μM) reduced proliferation, migration, and invasion. Reporter and overexpression experiments supported involvement of the miR-3677-5p/CXCL12/CXCR4 axis (Ma et al., 2022). Nevertheless, the high concentrations and absence of orthotopic *in vivo* validation limit conclusions regarding leukemic trafficking or extramedullary infiltration. In P-gp-overexpressing CEM/VLB1000 cells, 20(S)-Rg3, 20(R)-Rg3, 20(S)-PPD, and 20(S)-PPT increased intracellular daunorubicin accumulation (Chen et al., 2026). This finding is consistent with modulation of drug efflux but does not by itself establish reversal of multidrug resistance or improved therapeutic selectivity. Overall, current evidence supports effects on stem-like phenotypes, host-associated endpoints, migration-related signaling, and drug accumulation, but not direct eradication of functional LSCs or causal remodeling of the leukemic niche.

The context-dependent pharmacological effects reported across non-malignant and malignant hematopoietic models are summarized in Figure 1.

**FIGURE 1:**
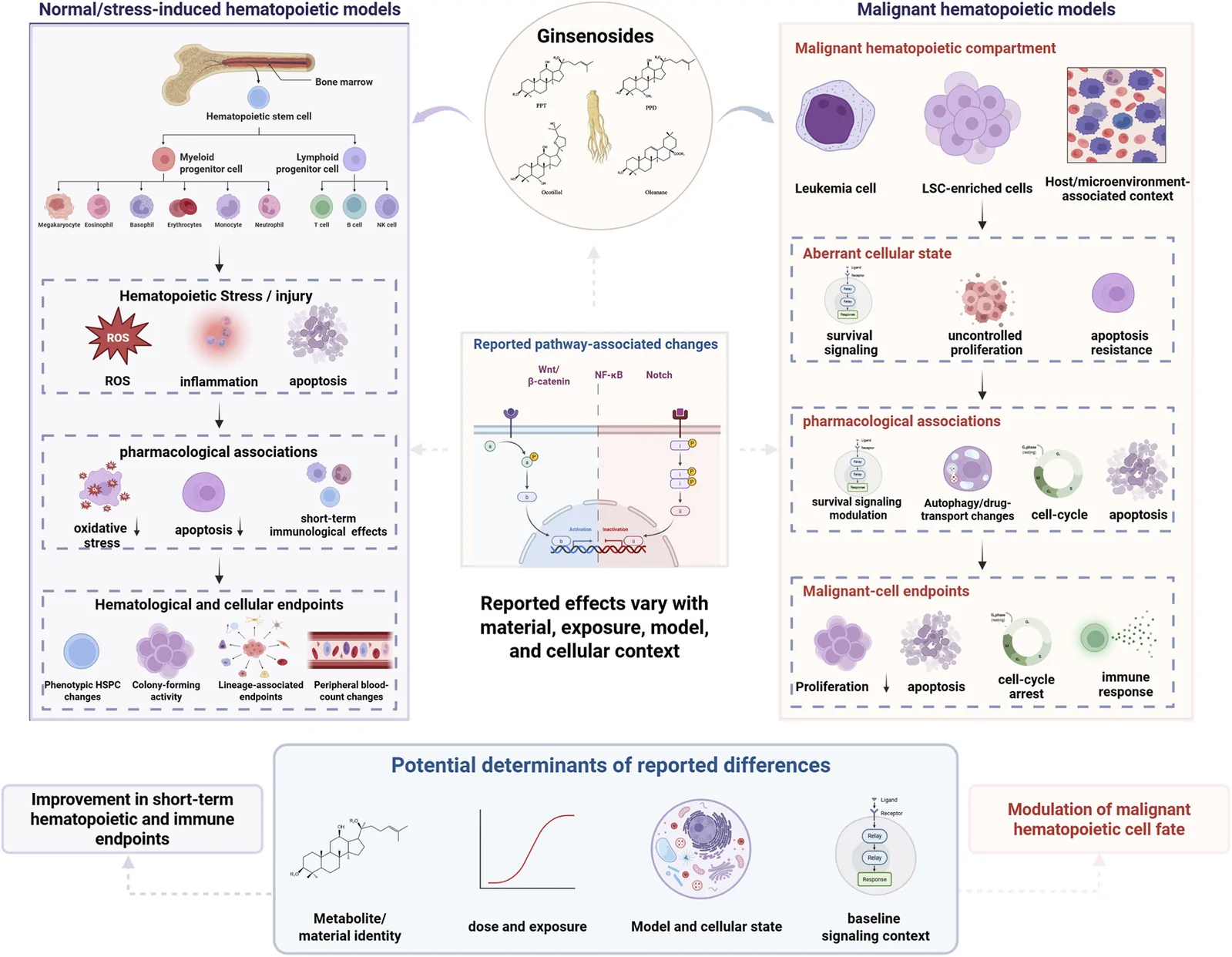
Context-dependent pharmacological effects of ginsenosides in hematopoietic models. Reported effects include short-term hematopoietic and immune changes in normal/stress models and altered proliferation, apoptosis, differentiation, autophagy, or drug transport in malignant models. These responses vary with material, exposure, model, and cellular context and do not establish clinical benefit or a therapeutic window.

## 3 Translational limitations

### 3.1 Pharmacokinetics, microbial biotransformation, and exposure

Absolute oral bioavailability remains poorly characterized in humans; available oral studies instead indicate low, delayed, and variable plasma exposure. Following oral administration of characterized red ginseng products, compound K, Rh2, PPD, and PPT were detected despite being absent from the administered products, consistent with intestinal deglycosylation, whereas Rh1 was not consistently detected (Tawab et al., 2003; Kim, 2013; Jin et al., 2019; Kim et al., 2020; Kim, 2018). Compound K exposure also correlated with fecal compound K-forming activity, supporting interindividual microbiota-related variability (Kim et al., 2020). Pharmacokinetic profiles differed between 20(S) and 20(R) stereoisomers and across experimental species (Bae et al., 2014; Jeon et al., 2020). Bone-marrow exposure has not been reported. Moreover, the micromolar concentrations used in several leukemia-cell studies exceeded the nanomolar plasma exposures reported after oral administration, although comparisons are limited by differences in formulation, administration route, protein binding, and exposure duration. Human exposure to 20(S)-Rg3 has been quantified after intramuscular, but not equivalent oral, administration (Zhao et al., 2016). Therefore, a clinically relevant therapeutic window remains undefined.

### 3.2 Safety and drug-interaction considerations

Formal toxicological assessment of purified ginsenosides and defined saponin fractions remains limited in hematological populations. The absence of overt toxicity in short-term cell or animal studies should not be interpreted as evidence of clinical safety, because chronic toxicity, marrow-specific toxicity, and exposure-dependent effects have rarely been evaluated.

Several purified ginsenosides, including Rs3, Rg5, 20(S)-Rg3, and Rg1, inhibited agonist-induced aggregation in isolated human platelets, accompanied by changes in GPVI/Syk-related signaling, intracellular Ca^2+^ mobilization, or cyclic-nucleotide pathways (Shin et al., 2026; Akram et al., 2025; Kwon, 2018; Zhou et al., 2014). Rg1, Rg5, and Rs3 also attenuated experimental arterial thrombosis. Platelet count and platelet function should therefore be evaluated separately. Although the clinical bleeding risk remains undefined, these findings warrant caution in thrombocytopenic or bleeding-prone patients and during concomitant anticoagulant or antiplatelet therapy.

Clinical evidence for ginseng–drug interactions remains limited and product-dependent. American ginseng reduced warfarin exposure and INR in one randomized trial, whereas studies of Asian or Korean ginseng products found no significant effect (Yuan et al., 2004; Jiang et al., 2004; Lee et al., 2008; Lee et al., 2010). Because these studies evaluated botanical preparations rather than purified ginsenosides, their findings cannot be attributed to a specific metabolite; nevertheless, INR monitoring is prudent during concomitant use. CK, PPD, PPT, and 20(S)-Rh2 inhibited P-gp in preclinical models, whereas human probe studies showed either no change or a modest, product-dependent increase in fexofenadine exposure (Malati et al., 2012; Kim et al., 2016; Li et al., 2014; Zhang et al., 2010). These metabolites also inhibited CYP2C9 and/or CYP3A4 *in vitro*, but corresponding human effects were absent or small (Malati et al., 2012; Kim et al., 2016; Liu et al., 2006). No dedicated pharmacokinetic studies have evaluated interactions with cyclosporine, androgens, or chemotherapy; therefore, the available CAA combination studies demonstrate concomitant use but do not establish interaction safety (Jiang et al., 2022; Kuang et al., 2016).

### 3.3 Clinical evidence and risk of bias

The therapeutic potential of ginsenosides in hematological disorders has been extensively investigated in preclinical studies, demonstrating hematopoietic regulatory, immunomodulatory, and protective activities. However, clinical investigations of ginsenoside-based interventions in hematological disorders remain relatively limited, and their clinical translation is still at an early stage.

Direct clinical evidence is limited to two small studies in chronic aplastic anemia (CAA). In a 24-week multicenter study, 79 patients were randomized by a random-number table to receive PNDC plus cyclosporine and reduced-dose testosterone undecanoate or cyclosporine plus full-dose testosterone undecanoate (Jiang et al., 2022). Among 78 evaluable patients, protocol-defined hematological response rates were 88.1% and 77.8%, respectively (P > 0.05), and endpoint blood counts were comparable. Female masculinization scores were lower in the PNDC/reduced-androgen group (P < 0.01). However, blinding, allocation concealment, and prospective registration were not reported, and the simultaneous reduction in androgen dose prevents attribution of this difference specifically to PNDC.

An earlier non-randomized study provided only supplementary evidence: 41 patients were enrolled, 36 completed 6 months of treatment, no PND-free concurrent control was included, and only three evaluable patients received PND alone (Kuang et al., 2016). Both studies used preparations reported to contain 92.44% total panaxadiol saponins by HPLC, but individual metabolite identities and contents were incompletely reported. These design and standardization limitations preclude conclusions regarding an independent hematological, androgen-sparing, or tolerability benefit of PND. Design and risk-of-bias details of the CAA studies are provided in Supplementary Table S2.

Indirect clinical evidence is provided by a meta-analysis of 18 randomized trials involving 2,222 patients with solid tumors receiving chemotherapy (Pan et al., 2020). Adjunctive Rg3 was associated with lower reported risks of leukocyte, hemoglobin, platelet, and neutrophil reductions. However, the underlying trials were predominantly small, methodologically weak, and heterogeneous in chemotherapy regimens and product reporting. These findings therefore do not establish a clinical benefit in primary hematological disorders.

Overall, current clinical evidence remains insufficient to corroborate the pharmacological effects reported in preclinical models.

## 4 Conclusion

Preclinical studies report context-dependent pharmacological effects of ginsenosides in normal and malignant hematopoietic settings. In non-malignant models, selected purified metabolites and defined saponin fractions were associated with changes in phenotypic HSPC, colony-forming, peripheral-blood, or immune endpoints. However, these findings generally do not establish durable HSC self-renewal because competitive transplantation and long-term multilineage reconstitution were rarely assessed. In malignant models, several ginsenosides induced apoptosis, cell-cycle arrest, differentiation-associated changes, or altered drug accumulation, but most studies relied on established cell lines, high micromolar concentrations, and limited normal-cell comparisons.

The apparent normal-protective/malignant-cytotoxic pattern should therefore be regarded as a context-dependent working hypothesis rather than demonstrated biological selectivity. Its determinants may include metabolite identity and stereochemistry, exposure, cellular state, disease model, and the basal activity of the affected pathway. Translation is further constrained by incomplete material standardization, variable microbial biotransformation, uncertain bone-marrow exposure, the absence of a defined therapeutic window, and unresolved platelet and drug-interaction risks. Direct clinical evidence in hematological disorders remains limited and methodologically limited. Future studies should use chemically defined preparations, clinically relevant exposures, normal and malignant hematopoietic comparators, causal mechanistic designs, and appropriately controlled clinical protocols before reproducible their clinical relevance can be determined.
